# Supplementary material for: Isolation and Identification of a TaTDR-Like Wheat Gene Encoding a bHLH Domain Protein, Which Negatively Regulates Chlorophyll Biosynthesis in Arabidopsis
Source: Int J Mol Sci. 2020 Jan 17;21(2):629. doi: 10.3390/ijms21020629 (PMC7014150; doi:10.3390/ijms21020629)
Supplement: Supplementary file 1 [file ijms-21-00629-s001.zip › ijms-665436-SI-to conversion/Supplementary Figure S1-S6.pdf]

Supplementary Figures:

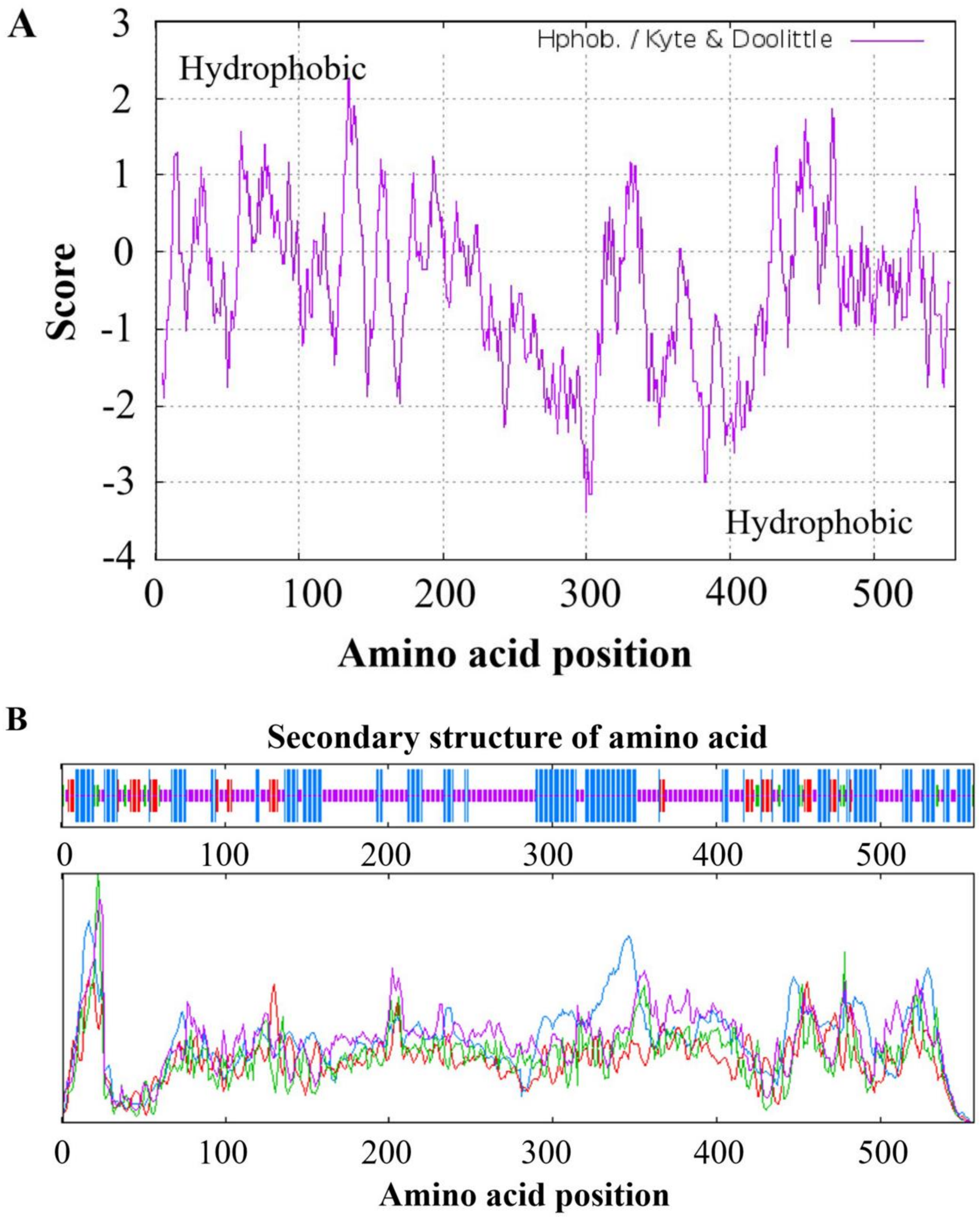

Figure S1. Hydropathy and Secondary structure analysis of TaTDRL protein. (**A**) Hydropathy analysis for the TaTDRL protein. (**B**) Secondary structure of wheat TaTDRL protein. The blue, red, green, and purple lines represent a-helix, extended strand, b-turn, and random coil regions, respectively.

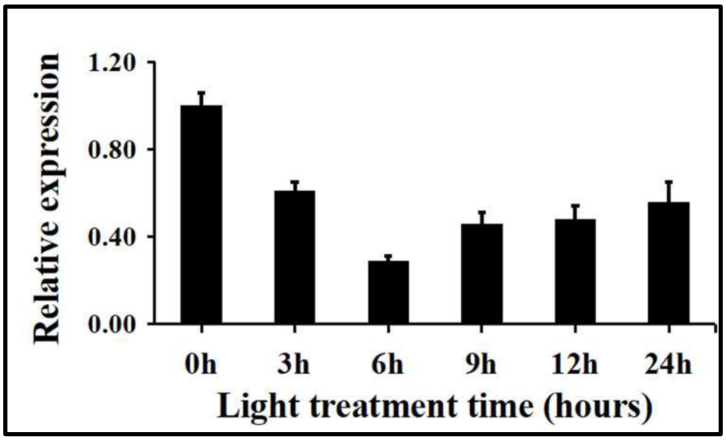

Figure S2. qRT-PCR analysis of TaTDRL expression in leaves of wheat cultivar XN1376 in response to light treatment.

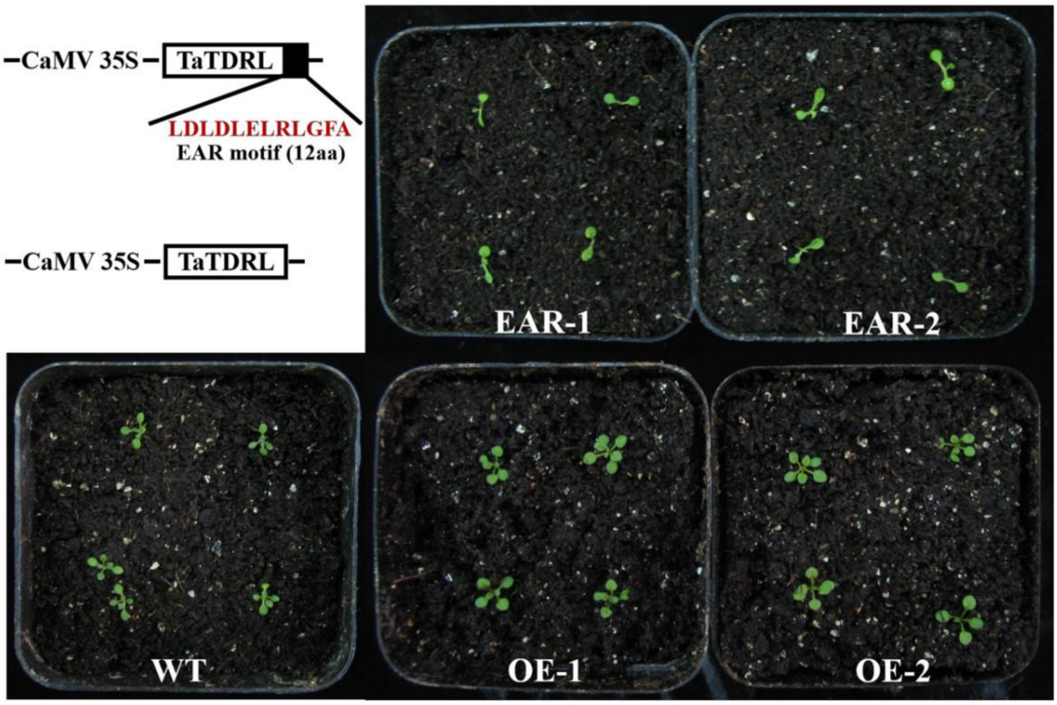

Figure S3. Schematic representation of the construct used for expression of the chimeric repressors, showing the amino acid sequences of the EAR-motif (LDLDLELRLGFA) repression domains. CaMV35S, cauliflower mosaic virus 35S promoter. Phenotypic characterization of Wild-type (WT) Arabidopsis, TaTDRL-OE and TaTDRL-EAR transgenic Arabidopsis plants.

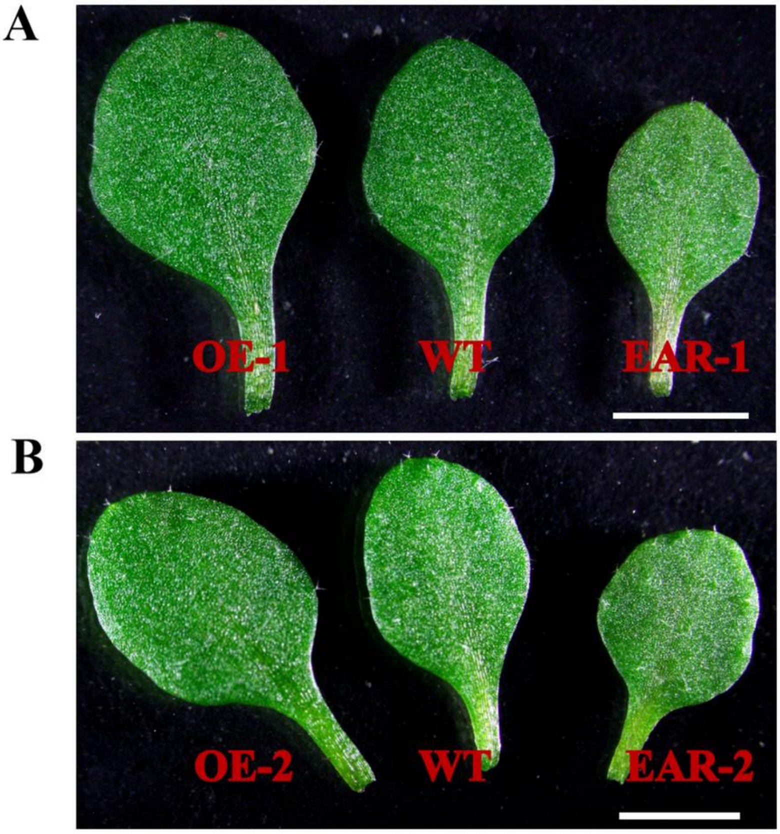

Figure S4. Leaf size of one-week-old transgenic and control plants. Bar = 2000 μm.

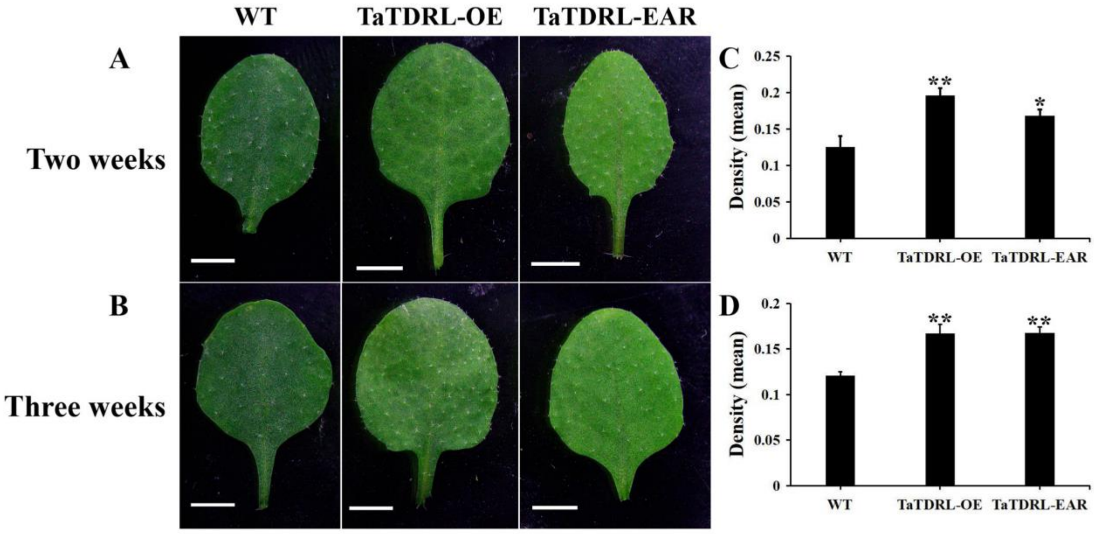

Figure S5. Further phenotypic analysis of TaTDRL transgenic lines and WT. (**A**) Phenotype of WT and transgenic leaves after two weeks of growth (**B**) Phenotype of WT and transgenic leaves after three weeks of growth (**C**) Density (mean) of two-week-old WT and transgenic leaves (n = 10). (**D**) Density (mean) of three-week-old WT and transgenic leaves (n = 10). Leaf color was quantitatively analyzed using image J. Density (mean) is the ratio of IOD (Integral optical density) to leaf area. Bars = 1 mm. Error bars represent means ± SD. Significant differences were tested using Student's test (* $p < 0.05$, ** $p <0.01$).

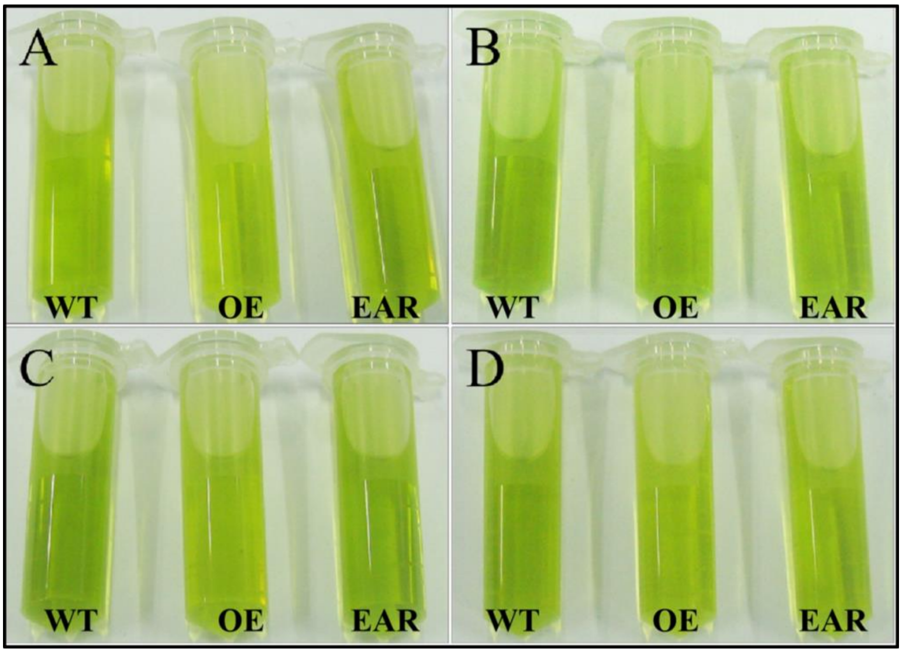

Figure S6. The images show the color changes of the chlorophyll in different time point between WT and transgenic plants. (**A**) After one week growth; (**B**) After two weeks growth; (**C**) After three weeks growth; (**D**) After four weeks growth.
