# Supplementary material for: Isolation and Identification of a TaTDR-Like Wheat Gene Encoding a bHLH Domain Protein, Which Negatively Regulates Chlorophyll Biosynthesis in Arabidopsis
Source: Int J Mol Sci. 2020 Jan 17;21(2):629. doi: 10.3390/ijms21020629 (PMC7014150; doi:10.3390/ijms21020629)
Supplement: Supplementary file 1 [file ijms-21-00629-s001.zip › ijms-665436-SI-to conversion/Supplementary Table S1.pdf]

Supplementary Table S1:

Table S1. The primers used in the study.

Primers for amplifying the TaTDRL

Primer Name	Primer Sequence
TaTDRL-P1-F	5′-ATGGGAGGAGGAGATTATCACC-3′
TaTDRL-P1-R	5′-TCAATCCATGGCGAGGTACTGC-3′
TaTDRL-P2-F	5′-CAATTAGGCACTTGCATTTCTG-3′
TaTDRL-P2-R	5′-GAAACACTTTTCGGTAGTGATG-3′

Primers for real-time PCR

Primer Name	Primer Sequence
TaActin-F	5′-TGTTGTTCTCAGTGGAGGTTCT-3′
TaActin-R	5′-CTGTATTTCCTTTCAGGTGGTG-3′
TaTDRL-qpcr-F	5′-CAACGACCGCCTCTACAAG-3′
TaTDRL-qpcr-R	5′-GCTCCTTCACCTGCTTCTG-3′
AtActin-F	5′-GGTAACATTGTGCTCAGTGGTGG-3′
AtActin-R	5′-AACGACCTTAATCTTCATGCTGC-3′
AtPORC-qpcr-F	5′-TTAGCAGACACAGGGAAATGG-3′
AtPORC-qpcr-R	5′-CAAGCGAAGCAAGATCAAGATG-3′

Primers for transcriptional activation in the yeast GAL4 system

Primer name	Primer sequence
TaTDRL-BD-F	5′-GCCATGGAGGCCGAATTCATGGGAGGAGGAGATTATCACC-3′
TaTDRL-BD-R	5′-CGGCCGCTGCAGGTCGACTCAATCCATGGCGAGGTACTGC-3′
TaTDRL-NBD-F	5′-GCCATGGAGGCCGAATTCATGGGAGGAGGAGATTATCACC-3′
TaTDRL-NBD-R	5′-CGGCCGCTGCAGGTCGACGTTCTTGCACTGCTGCCGCTTC-3′
TaTDRL-HLHBD-F	5′-GCCATGGAGGCCGAATTCCTCGAGGCGGAGCGGAAGCGGC-3′
TaTDRL-HLHBD-R	5′-CGGCCGCTGCAGGTCGACCTCCTTCACCTGCTTCTGCAGC-3′
TaTDRL-CBD-F	5′-GCCATGGAGGCCGAATTCCTGCAGGACGAGCTGGAGGACC-3′
TaTDRL-CBD-R	5′-CGGCCGCTGCAGGTCGACTCAATCCATGGCGAGGTACTGC-3′
TaTDRL-NHLHBD-F	5′-GCCATGGAGGCCGAATTCATGGGAGGAGGAGATTATCACC-3′
TaTDRL-NHLHBD-R	5′-CGGCCGCTGCAGGTCGACCTCCTTCACCTGCTTCTGCAGC-3′
TaTDRL-HLHCBD-F	5′-GCCATGGAGGCCGAATTCCTCGAGGCGGAGCGGAAGCGGC-3′
TaTDRL-HLHCBD-R	5′-CGGCCGCTGCAGGTCGACTCAATCCATGGCGAGGTACTGC-3′
TaTDRL-EGFP-F	5′-CGAGCTCAAGCTTCGAAATGGGAGGAGGAGATTATCACC-3′
TaTDRL-EGFP-R	5′-CGACTGCAGAATTCGAAATCCATGGCGAGGTACTGCAG-3′

Primers for yeast two-hybrid

Primer name	Primer sequence

TaPIF1-AD-F	5′-CCATGGAGGCCAGTGAATTCATGAGCCAATTCGTGCCAGATT-3′
TaPIF1-AD-R	5′-AGCTCGAGCTCGATGGATCCTCATCTTTTGTTTGGGTGTATC-3′
AtPIF1-AD-F	5′-CCATGGAGGCCAGTGAATTCATGCATCATTTTGTCCCTGACT-3′
AtPIF1-AD-R	5′-AGCTCGAGCTCGATGGATCCTTAACCTGTTGTGTGGTTTCCG-3′
TaTDRL-CBD-F	5′-GCCATGGAGGCCGAATTCCTGCAGGACGAGCTGGAGGACC-3′
TaTDRL-CBD-R	5′-CGGCCGCTGCAGGTCGACTCAATCCATGGCGAGGTACTGC-3′

Primers for bimolecular fluorescence complementation assays

Primer name	Primer sequence
AtPIF1-cEYFP-F	5′-CGAGCTCAAGCTTCGAAATGCATCATTTTGTCCCTGACT-3′
AtPIF1-cEYFP-R	5′-CGACTGCAGAATTCGAATTAACCTGTTGTGTGGTTTCCG-3′
TaPIF1-cEYFP-F	5′-CGAGCTCAAGCTTCGAAATGAGCCAATTCGTGCCAGATT-3′
TaPIF1-cEYFP-R	5′-CGACTGCAGAATTCGAATCATCTTTTGTTTGGGTGTATC-3′
TaTDRL-nEYFP-F	5′-CGAGCTCAAGCTTCGAAATGGGAGGAGGAGATTATCACC-3′
TaTDRL-nEYFP-R	5′-CGACTGCAGAATTCGAAATCCATGGCGAGGTACTGCAG-3′

For construction of a transgene vector

Primer name	Primer sequence
TaTDRL-OE-F	5′-GGGGACTCTTGACCATGGAGATGGGAGGAGGAGATTAT-3′
TaTDRL-OE-R	5′-TTCTTCTCCTTTACTAGTTTACTTATCGTCGTCATCCTTGTAATCATCCATGGCGAGGTA-3′
TaTDRL-EAR-F	5′-GGGGACTCTTGACCATGGAGATGGGAGGAGGAGATTAT-3′
TaTDRL-EAR-R	5′-TTCTTCTCCTTTACTAGTTTAAGCGAAACCCAAACGGAGTTCTAGATCCAGATCCAGATCCATGGCGAGGTA-3′
