# Supplementary material for: Isolation and Identification of a TaTDR-Like Wheat Gene Encoding a bHLH Domain Protein, Which Negatively Regulates Chlorophyll Biosynthesis in Arabidopsis
Source: Int J Mol Sci. 2020 Jan 17;21(2):629. doi: 10.3390/ijms21020629 (PMC7014150; doi:10.3390/ijms21020629)
Supplement: Supplementary file 1 [file ijms-21-00629-s001.zip › ijms-665436-SI-to conversion/Supplementary Table S2.pdf]

Supplementary Table S2:

Table S2. Cis-elements in the upstream regulation region of *TaTDRL* gene.

Associated putative function	Cis-elements in the promoter region
Abscisic acid responsiveness	ABRE
salicylic responsiveness	TCA-element
MeJA-responsibeness	CGTCA-motif, TGACG-motif
Gibberellin-responsive	GARE-motif
Essential for the anaerobic induction	ARE
Auxin responsiveness	AuxRR-core
Meristem expression	CAT-box
MYBHv1 binding site	CCAAT-box
Anoxic specific inducibility	GC-motif
Low-temperature responsiveness	LTR
drought-inducibility	MBS
Zein metabolism regulation	O2-site
Seed-specific regulation	RY-element
Light responsive element	AE-box, Box4, GATA-motif, GT1-motif, GTGGC-motif, L-box, Sp1 (3), TCT-motif, G-box (5), G-Box
